# Swallowing Assessment in Parkinson’s Disease: Patient and Investigator Reported Outcome Measures are not Aligned

**DOI:** 10.1007/s00455-020-10201-3

**Published:** 2020-10-31

**Authors:** M. R. A. van Hooren, R. Vos, M. G. M. H. Florie, W. Pilz, B. Kremer, L. W. J. Baijens

**Affiliations:** 1 Department of Otorhinolaryngology and Head and Neck Surgery, Maastricht University Medical Centre, Maastricht, The Netherlands; 2 Department of Otorhinolaryngology and Head and Neck Surgery, Zuyderland Medical Centre, PO Box 5500, 6130 MB Heerlen, Sittard-Geleen, The Netherlands; 3 Department of Methodology and Statistics, Maastricht University, Maastricht, The Netherlands; 4 Care and Public Health Research Institute – CAPHRI, Maastricht University Medical Center, Maastricht, The Netherlands; 5 School for Mental Health and Neuroscience – MHeNs, Maastricht University Medical Center, Maastricht, The Netherlands

**Keywords:** Oropharyngeal dysphagia, Parkinson's disease, Patient reported outcome measures, Investigator reported outcome measures, Neural network analysis

## Abstract

This study determines the relationship between patient and investigator reported outcome measures (PROMs versus IROMs) on oropharyngeal dysphagia (OD) in Parkinson’s disease (PD). The PROMs used are the MD Anderson Dysphagia Inventory (MDADI) and the Dysphagia Severity Scale (DSS). The IROMs used are fiberoptic endoscopic evaluation of swallowing (FEES) and videofluoroscopy of swallowing (VFS). Ninety dysphagic PD patients were included. Multilayer perceptron (MLP) neural network analysis was used to investigate the relationship between PROMs and IROMs on OD in PD. MLP neural network analysis showed a moderate agreement between PROMs and IROMs, with an area under the curve between 0.6 and 0.7. Two-step cluster analysis revealed several clusters of patients with similar scores on FEES and/or VFS variables, but with significant different scores on MDADI and DSS variables. This study highlights that there are PD patients with similar FEES and/or VFS findings that cannot be lumped together under the same pathophysiological umbrella due to their differences in PROMs. Since the exact origin of these differences is not fully understood, it seems appropriate for the time being to take into account the different dimensions of OD during the swallowing assessment so that they can be included in a patient-tailored treatment plan.

## Introduction

Oropharyngeal dysphagia (OD) is a common non-motor symptom in idiopathic Parkinson’s disease (PD) [1, 2]. The burden of OD in PD is immense, as it affects health-related quality of life (QoL) [3–5], and may lead to complications such as aspiration pneumonia [6, 7]. Even in an early PD stage, patients report a lower swallow-related QoL compared to healthy control subjects [3]. However, patients with a moderately advanced PD disease stage do not report the worst swallow-related QoL compared to the patients in the early disease stages.[3]. It appears that the swallow-related QoL only decreases further when the patients are in advanced Hoehn and Yahr (H&Y) stage [3]. This suggests that the decline of self-report swallow-related QoL stagnates despite the progression of PD during its early H&Y stages or that PD patients develop compensatory swallowing strategies or coping mechanisms that inhibit the decline of swallow-related QoL despite the progression of OD. In case of the latter, an inconsistency is expected between the patient reported outcome measures (PROMs) and the investigator reported outcome measures (IROMs) on swallowing.

The inconsistency between patient self-report swallow-related QoL and the actual swallowing function using fiberoptic/flexible endoscopic evaluation of swallowing (FEES) or videofluoroscopy of swallowing (VFS) has been described in several studies on OD due to other underlying disorders such as acute stroke, myotonic dystrophy and head and neck cancer [8–11]. Silent aspiration occurs in about 20% of the PD patients and is one of the main risk factors for developing aspiration pneumonia [12, 13]. For PD, the relationship between the results of validated self-report swallow-related QoL questionnaires and of instrumental tools such as FEES or VFS has not been reported in the literature before.

Therefore, the objective of the present study was to determine the relationship between PROMs and IROMs on swallowing in PD patients. To further explore the characteristics of this relationship, clinically relevant subgroups of patients within the study population, based on similar PROMs and/or IROMs, were identified and studied.

## Materials and Methods

### Participants

PD patients with dysphagic complaints were recruited from all over the Netherlands between 2007 and 2011. A neurologist clinically diagnosed the PD according to the UK Parkinson’s Disease Society Brain Bank and the H&Y scoring system [14, 15]. The majority of the patients was referred by their speech and language pathologist (SLP) who had identified clinically relevant symptoms of OD during a clinical swallowing examination. Individuals were enrolled in the study if they were in a stable period of PD (periods without large fluctuations, especially in motor function). The exclusion criteria were: being older than 85 years (presbyphagia); having had speech therapy during the previous six months (benefit of treatment and attention); scoring below 23 on a Mini Mental State Examination (MMSE) [16]; suffering from severe depression or having a psychiatric diagnosis; not being able to speak Dutch; being illiterate or blind; having a history of stroke, and having the antiparkinsonian medication regimen changed within the past six weeks. Also, patients with a history of extensive surgery or cancer of the head and neck region were excluded. Written informed consent was obtained from all patients and the medical ethics committee (MEC) approved the study protocol (MEC 05-237).

### Evaluation Protocol

All patients underwent a standardized examination protocol in the same tertiary referral university hospital by the same multidisciplinary team in order to guarantee standardized data collection. The examination protocol included an otorhinolaryngological examination, done by a laryngologist, checking the integrity of the cranial nerves and the upper aerodigestive tract; the MMSE; FEES; VFS; and patient self-report swallow-related QoL questionnaires, namely the MDADI and DSS. All examinations and questionnaires were performed at the same day during the 'on' motor phase (within 90–120 min after intake of antiparkinsonian medication) [17].

The MDADI is a self-administered, psychometrically validated OD-specific questionnaire. It is designed to assess the impact of OD on health-related QoL, although some MDADI items are also related to functional health status (FHS) [18–20]. For the current study the validated Dutch MDADI version for neurogenic OD was used [21]. Like the original English version, the validated Dutch translation of the MDADI consists of 20 items pooled in 4 subscales: the global scale (1 item); the functional scale (5 items); the physical scale (8 items); and the emotional scale (6 items) [21, 22]. The global assessment question (MDADI-G) evaluates the effect of OD on overall QoL. The functional scale (MDADI-F) illustrates the impact of OD on daily activities. The physical scale (MDADI-P) measures the patient’s self-perception of the physical impact of OD. The emotional scale (MDADI-E) represents the patient’s affective response to the swallowing disorder in terms of embarrassment, self-esteem, and self-consciousness. All items are scored on a 5-point scale (1–5), where ‘1’ corresponds to ‘total agreement’ and ‘5’ to ‘total disagreement’. Responses on all domains were summed to calculate the MDADI total score (MDADI-T). The minimum score is 20, indicating poor functioning, and the maximum possible score is 100.

The DSS is a visual analogue scale that was used to assess patient’s perception of the severity of the swallowing impairment on the day of the examination [23]. The DSS score ranges from 0 (extreme swallowing impairment or inability to swallow) to 100 mm (no swallowing impairment).

The FEES examinations were performed by an experienced laryngologist together with the SLP. First, patients had to perform three swallows of 10 cc thin liquid (water) followed by three swallows of 10 cc thick liquid (applesauce—One 2 fruit^®^) and three bite-sized crackers (Delhaize mini toast 80 gr^®^). All liquids were dyed with 5% methylene blue (10 mg/ml). The viscosity of the liquid bolus consistencies was measured at 25 °C and 50 s^−1^ of shear rate resulting in 1 mPa·s for thin liquid (International Dysphagia Diet Standardization Initiative (IDDSI) level 0) and 1200 mPa s for thick liquid (IDDSI level 3) [24]. A flexible fiberoptic endoscope, Pentax FNL-10RP3 (Pentax Canada Inc., Mississauga, Ontario, Canada), was used during the FEES examination. The tip of the endoscope was in ‘high position’, just above the epiglottis, where the scope could not interfere with closure of the laryngeal vestibule [25]. FEES images were obtained using an Alphatron Stroboview ACLS camera, Alphatron Lightsource, IVACX computerized video archiving system (Alphatron Medical Systems, Rotterdam, The Netherlands), and recorded on a DVD. No topical anesthetic or nasal vasoconstrictor was used during the exam.

During the VFS, patients were offered three trials of thin liquid low-density barium 40% weight/volume (Micropaque suspension^®^ 1000 g/l) and three trials of thick liquid (50 cc applesauce—One 2 fruit^®^ + 150 g barium powder – E-Z-HD^®^ 984.5 mg/g powder for oral suspension) followed by three bite-sized crackers (Delhaize mini toast 80 gr^®^) coated with barium paste. The viscosity of the liquid bolus consistencies was measured at 25 °C and 50 s^−1^ of shear rate resulting in 12.05 mPa s for thin liquid (IDDSI level 0) and 1900 mPa s for thick liquid (IDDSI level 4) [24]. Similar to what was done during the FEES examination, each participant swallowed the bolus consistencies upon command and in the same sequence (thin liquid, thick liquid, and bite-sized cracker). The field of the videofluoroscopic image included the lips, the oral cavity, the cervical spine, and the proximal cervical esophagus (in lateral position; dental prosthesis in position). Videofluoroscopic images were obtained with a Philips Diagnost 97 system (Philips Medical Systems, Eindhoven, The Netherlands) and recorded at twenty-five frames per second using a mini-DV camera-recorder Panasonic AG-DVC30 (Matsushita Electric Industrial Co., Osaka, Japan).

For each FEES and VFS swallow visuoperceptual ordinal variables (Table 1) were scored at varying speed (slow motion, normal, frame-by-frame speed) by two observers who followed a training program described in previous studies [18, 26]. The observers were blinded to patient identity, medical history, and to each other’s rating scores (independent rating). Each observer was asked to limit the evaluation period to a maximum of 2 h in order to maintain optimal attention and reduce fatigue-related bias.

**Table 1:** Definition and ordinal scale of the visuoperceptual fiberoptic/flexible endoscopic evaluation of swallowing (FEES) and/or videofluoroscopy of swallowing (VFS) variables

Variable	Definition	Scale
Piecemeal deglutition^a^ (FEES and VFS)	Sequential swallowing on the same bolus	0 = 1 swallow, no additional swallows 1 = 1 swallow with 1 additional swallow 2 = 1 swallow with 2 additional swallows 3 = 1 swallow with 3 additional swallows 4 = 1 swallow with 4 or more additional swallows
Preswallow posterior spill^a^ (FEES)	Preswallow loss of bolus into the pharynx	0 = no posterior spill 1 = trace 2 = more than trace, but less than 50% 3 = > 50% of the bolus 4 = whole bolus flows into the pharynx without swallowing
Postswallow vallecular pooling (FEES and VFS)	Pooling in the vallecula after the swallow	0 = no pooling 1 = mild to moderate pooling (filling of less than 50% of the vallecula) 2 = severe pooling (filling of more than 50% of the vallecula up to complete filling)
Postswallow pyriform sinus pooling (FEES and VFS)	Pooling in the pyriform sinuses after the swallow	0 = no pooling 1 = mild to moderate pooling (filling of less than 50% of the pyriform sinuses) 2 = severe pooling (filling of more than 50% of the pyriform sinuses up to complete filling)
Penetration aspiration scale (FEES and VFS)	Penetration and/or aspiration according to the Rosenbek scale [30]	8-point scale [30]

^a^Not scored for bite-sized cracker, since piecemeal deglutition and preswallow posterior spill can be normal aspects of swallowing during solid bolus processing/mastication

### Statistical Analysis

#### Observer Agreement Analysis

Observer agreement analysis was performed using a weighted kappa index of agreement (intraobserver and interobserver) for all visuoperceptual ordinal FEES and VFS variables.

#### Neural Network Analysis

To elaborate the clinically complex relationship between PROMs and IROMs on OD in PD, a multilayer perceptron (MLP) neural network analysis was used. An MLP neural network analysis is a relatively modern statistical technique to process complex (non-)linear data. This statistical approach can be used for many purposes and is especially useful for data containing several kinds of variables (ordinal, binary, continuous, etc.). The most common purposes of MLP neural network analysis are pattern recognition, forecasting, and modeling of complex relationships between data [27]. In health care it has been used for several diagnostic purposes such as manometry for OD in PD [28]. To reach the objective of this study an MLP neural network, from now on referred as MLP, was used to model the complex relationship between PROMs, IROMs, and demographic patient characteristics. The MLP is composed of an input layer to receive the signal, an output layer that makes a decision or prediction about the input, and in between those two, an arbitrary number of ‘hidden’ layers that are the true computational engine of the MLP. Feed forward networks such as MLPs are like tennis. Your aim is to score a point. Every time you miss, you have to learn from your mistake to improve the next serve. You can think of this tennis of guesses and answers as a kind of accelerated science, since each guess is a test of what we think we know, and each response is feedback letting us know how wrong we are. So, to model the data, an MLP uses one or more ‘input nodes’, and one or more ‘output nodes’. In this study the input nodes were the PROMs (MDADI subscales and DSS) and demographic patient characteristics and the output nodes were the IROMs (FEES and VFS variables). The MLP technique was used to determine the relationship between each input node and output node resulting in a ‘hidden node’. This hidden node is a robust weight between each input and output node describing their relationship to each other. In this way multiple hidden nodes will be obtained. Besides the weights between the input and output nodes, the MLP determines the weight between the hidden nodes as well. These steps result in multiple layers of hidden nodes. Every input, output, and hidden node is therefore connected to each other resulting in a complex network of weights. By training this MLP, the robust weight will become more accurate. The purpose of training the MLP is to find the optimal combination of weights resulting in the smallest error. To train the neural network, a training set is used. The input data (PROMs and demographic patient characteristics) of the training set was offered one by one to the network. Based on the robust weights between the input, output, and hidden nodes the output data could be calculated. This PROMs output data can then be compared to the actual output data of the IROMs and the differences between these two were marked as error. Finally, this error was used to recalculate the weight between all input, output, and hidden nodes resulting in the smallest possible error. These steps of training were repeated several times in order to develop the MLP based on the input and output data with the smallest error. This form of training is called back propagation. In this study the training set comprised 70% of the samples chosen at random. The training steps were repeated 50 times and the results were averaged [27].

The extent to which the MLP can predict the output data from the input data can be visualized in a receiver operating characteristics curve (ROC-curve). In case of an adequate neural network and a sufficient agreement between the input and output data, the neural network will predict the outcome data correctly to a large extent based on the input data. This accuracy can be calculated with an area under the curve (AUC). A high AUC, means an adequate neural network and a strong relationship and agreement between the PROMs and IROMs [29].

Since several OD-related variables (MDADI, DSS, FEES, and VFS) and demographic patient-related variables (age, gender, and H&Y scale) were used, there was a high chance of having a missing value on one of them. A complete case analysis would tremendously decrease the population size of the study. Therefore, besides the complete case analysis, multiple imputation was performed in the MLP analysis using fully conditional specification to account for missing values. By using multiple imputation, the missing values were estimated within the standard error. These estimations were repeated 200 times. In this way, 90 patients could be included in the MLP analysis and 200 unique datasets were created to develop the MLP feed forward network. The complete case analysis was used to verify that the imputation was valid.

To improve statistical power in the MLP analysis, patients were divided in three clinical patient labels based on the FEES and VFS ordinal variable outcome: *glossopalatal*, *pooling* or *aspiration*. It was possible for one patient to have multiple clinical labels. Patients received the *glossopalatal* label if either their FEES and/or VFS exam was scored impaired (score of 1 or higher) on one or more of the following variables during one or more bolus consistencies: preswallow posterior spill and/or piecemeal deglutition. Likewise, patients were assigned to the clinical patient label *pooling* if their swallowing exam was scored impaired (score of 1 or higher) on postswallow vallecular and/or pyriform sinus pooling. The clinical patient label *aspiration* was assigned to patients presenting penetration and/or aspiration according to the penetration-aspiration scale by Rosenbek et al. [30]. If patients did not have an impaired score on these FEES and/or VFS variables, then they were used as a control group for this particular clinical patient label.

#### Two-Step Cluster Analysis

Usually patients are divided in groups based on known demographic characteristics such as gender or whether they have a disease or not. By using two-step cluster analysis, patients are divided in clusters based on all available data. It is a tool to find ‘hidden’ clusters or patterns within the multivariate data that otherwise would not be found. The goal of two-step cluster analysis is to categorize patients by minimizing the within-cluster variation and maximizing the between-cluster variation. This leads to homogenous ‘natural’ clusters of patients with similar characteristics based on the multivariate data. With these newly formed clusters, additional statistical analysis can be done [31].

To obtain a better insight into the possible characteristics of this relationship between PROMs and IROMs, a two-step cluster analysis was used to explore whether there are clusters of patients with similar outcomes on FEES or VFS resulting in similar clinical patient labels, but with different outcomes on MDADI or DSS scores. One-way analysis of variance F-overall tests for means (ANOVA) was used to determine the mean differences of the MDADI and DSS scores between the clusters of patients. Bonferroni post-hoc analysis was carried out to correct for multiple testing. A *p* value ≤ 0.05 was considered statistically significant. Fisher exact test was used to identify significant differences in demographic patient characteristics (age, gender, H&Y scale) between the different clusters within each clinical patient label. A complete case analysis was used for the two-step cluster analysis. All statistical analyses were performed using IBM SPSS Statistics for Windows, version 23 (IBM, Armonk, NY).

## Results

### Participants

This study included ninety PD patients with swallowing complaints, of which sixty-seven were male. The median age was 67 years (range: 42–82 years) and the median H&Y score was 2 (range: 1–5). All patients were on a total oral diet, although seven patients (8%) required a modified texture diet. The mean MDADI-T score for the total group was 69 and the mean DSS score was 68 (standard deviation 14 and 24, respectively). The duration of the PD since diagnosis was at least 5 years. The floor or ceiling effect was considered negligible as few patients got the lowest or highest possible score for MDADI-T and DSS (4% and 10%, respectively). All patients used levodopa except for seven (8%) of the ninety PD patients. These patients did not use any antiparkinsonian medication. Due to the small number of patients without levodopa use, this was not included in the Fisher exact test. However, care was taken to ensure that all measurements in patients on levodopa were performed during the 'on' motor phase. The “on–off” phenomenon in PD refers to a switch between mobility and immobility in levodopa-treated patients, which occurs as an end-of-dose worsening of motor function [17].

### Observer Agreement Analysis

All FEES and VFS variables had sufficient intra- and interobserver agreement (i.e., weighted Cohen’s kappa > 0.6) and further inferences were drawn based on the data of the observer with the highest intraobserver agreement scores.

### Multilayer Perceptron (MLP) Neural Network Method

Table 2 shows the mean AUC, and the 95% confidence interval (CI) per clinical patient label. The mean scores of the MDADI-T and DSS were determined per clinical patient label.

**Table 2:** Mean results of the neural network analysis per clinical patient label

Clinical patient label	Glossopalatal	Pooling	Aspiration
Number of patients^a^ (*n* = 90)	51	36	19
Mean MDADI-T (score range 20–100)	68	67	64
Mean DSS (score range 0–100)	67	67	60
Mean AUC^b^	0.64	0.65	0.70
95% CI	0.62–0.65	0.63–0.67	0.67–0.72

*MDADI-T* MD Anderson dysphagia inventory-total score, *DSS* Dysphagia Severity Scale, *AUC* area under the curve, *CI* confidence interval

^a^Patients can have more than one clinical patient label

^b^Calculation of AUC and 95% CI were obtained after multiple imputation and averaging of 50 runs of MLP analysis for all (*n* = 200) imputed datasets

### Two-Step Cluster Analysis

For each clinical patient label a two-step cluster analysis was performed to identify clusters of patients sharing similar outcomes on FEES or VFS, but with different outcomes on MDADI or DSS scores. This analysis revealed three new clusters of patients within the clinical patient label *glossopalatal*, two clusters for the clinical patient label *poolin*g, and three new clusters for the clinical patient label *aspiration*. Using Fisher exact test, no significant differences (*p* value ≥ 0.05) for confounders (age, gender, H&Y scale) were found between the different clusters of patients within each clinical patient label.

#### Clinical Patient Label Glossopalatal

For the clinical patient label *glossopalatal* three clusters of patients were found. A complete case analysis of seventy-eight patients was carried out for this clinical patient label. The mean MDADI subscale and DSS scores are listed in Table 3. Cluster 1 (33%; *n* = 26/78) and 2 (32%; *n* = 25/78) contain patients presenting preswallow posterior spill and/or piecemeal deglutition, and cluster 3 (35%; *n* = 27/78) consists of patients who did not present preswallow posterior spill and/or piecemeal deglutition during FEES and/or VFS.

**Table 3:** Means (95% CI) of the MDADI subscale and DSS scores for each patient cluster within the clinical patient labels

Cluster	Preswallow posterior spill and/or piecemeal deglutition		MDADI-F	MDADI-P	MDADI-E	DSS
*Glossopalatal*						
1	Impaired	Mean (95% CI)	17 (15–19)	22 (20–24)	17 (16–19)	48 (41–56)
2	Impaired	Mean (95% CI)	23 (22–24)	32 (30–34)	24 (23–25)	83 (77–89)
3	Normal	Mean (95% CI)	21 (19–22)	28 (25–30)	22 (20–23)	69 (60–78)
		*p* value	0.00	0.00	0.00	0.00

Cluster	Vallecular and/or pyriform sinus pooling		MDADI-F	MDADI-P	MDADI-E	DSS
*Pooling*						
1	Impaired	Mean (95% CI)	19 (19–20)	27 (26–28)	21 (20–21)	66 (64–70)
2	Normal	Mean (95% CI)	20 (20–21)	27 (26–28)	21 (20–22)	67 (65–72)
		*p* value	0.44	0.79	0.53	0.79

Cluster	Penetration and/or aspiration		MDADI-F	MDADI-P	MDADI-E	DSS
*Aspiration*						
1	Normal	Mean (95% CI)	22 (21–23)	29 (28–31)	23 (22–24)	73 (66–79)
2	Impaired	Mean (95% CI)	21 (19–22)	28 (25–30)	21 (19–23)	60 (49–71)
3	Normal	Mean (95% CI)	13 (12–12)	19 (16–22)	15 (13–17)	55 (41–69)
		*p* value	0.00	0.00	0.00	0.02

The mean difference of the MDADI and DSS scores between the clusters of patients was determined using the one-way analysis of variance *F*-overall test for means (ANOVA)

*CI* confidence interval, *DSS* Dysphagia Severity SCALE, *MDADI* MD Anderson Dysphagia inventory, *F* functional, *P* physical, *E* emotional

**Fig. 1:**
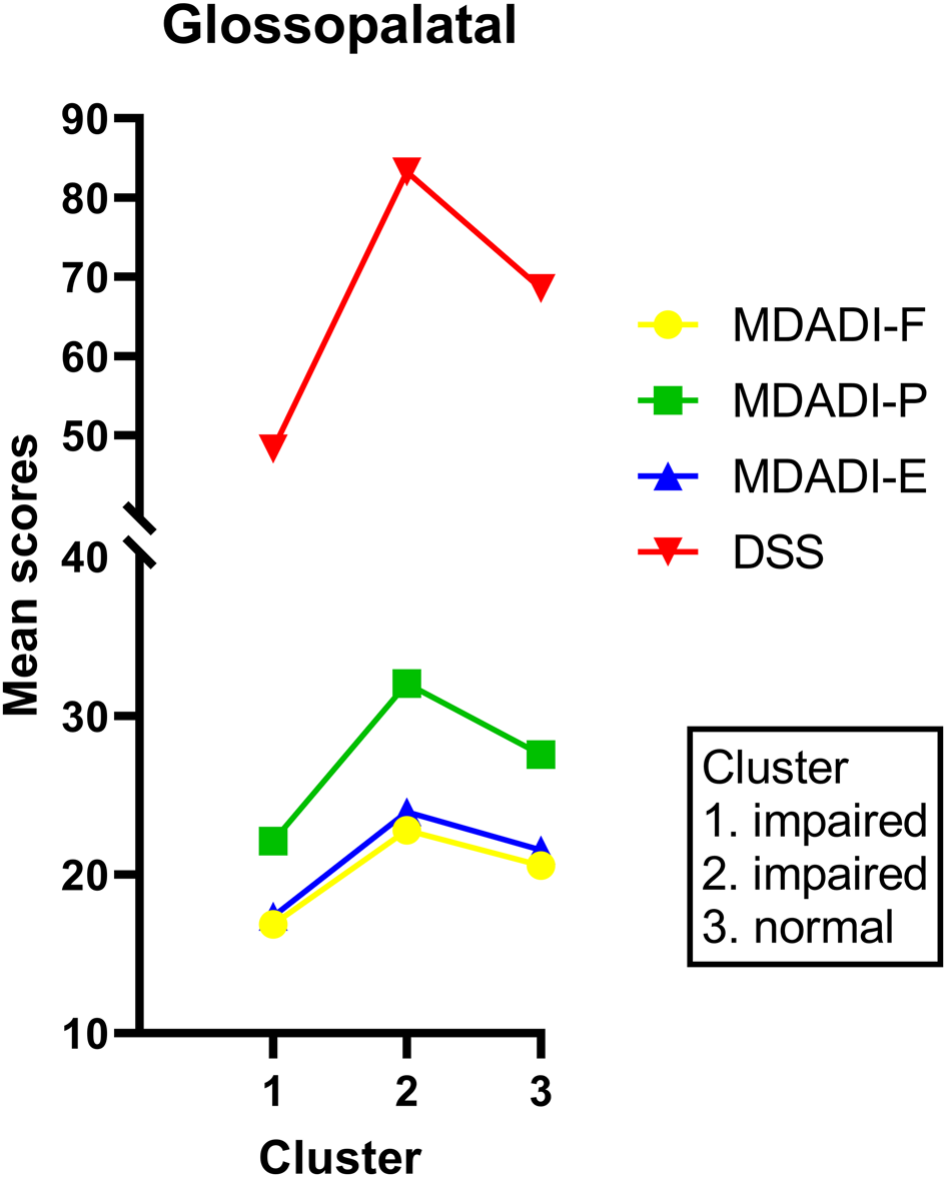
Mean MDADI subscale and DSS scores per patient cluster for the clinical patient label *glossopalatal* (presence of preswallow posterior spill and/or piecemeal deglutition)

The mean MDADI subscale and DSS scores per patient cluster are presented in Fig. 1. The mean MDADI subscale and DSS scores were significantly different (*p* < 0.001) between the two clusters of patients presenting preswallow posterior spill and/or piecemeal deglutition (cluster 1 and 2). Although patients in cluster 1 and 2 have similar scores on the IROMs, the mean scores of the PROMs of patients in cluster 2 were significantly higher (higher swallow-specific QoL) compared to patients in cluster 1 (Fig. 1). For the patient cluster without signs of preswallow posterior spill and/or piecemeal deglutition (cluster 3) the mean MDADI subscale and DSS scores were significantly higher (higher swallow-specific QoL) compared to cluster 1. However, cluster 2 and 3 showed similar mean PROMs scores. Only the mean MDADI-P and mean DSS score were significantly different between patients in cluster 2 and cluster 3 (*p* = 0.008 and *p* = 0.022 respectively). The mean MDADI-E and MDADI-F score did not significantly differ between cluster 2 and 3 (*p* = 0.088 and *p* = 0.052 respectively). It seems that although cluster 2 and 3 have different scores on the IROMs, their mean scores on the PROMs were fairly similar.

#### Clinical Patient Label Pooling

For the clinical patient label *pooling*, only two clusters of patients could be identified. Cluster 1 (46%; *n* = 36/78) contains patients presenting postswallow vallecular and/or postswallow pyriform sinus pooling and cluster 2 (54%; *n* = 42/78) consists of patients without these signs of OD. No significant differences in the mean MDADI subscale and DSS scores were found between both patient clusters (Table 3).

#### Clinical Patient Label Aspiration

**Fig. 2:**
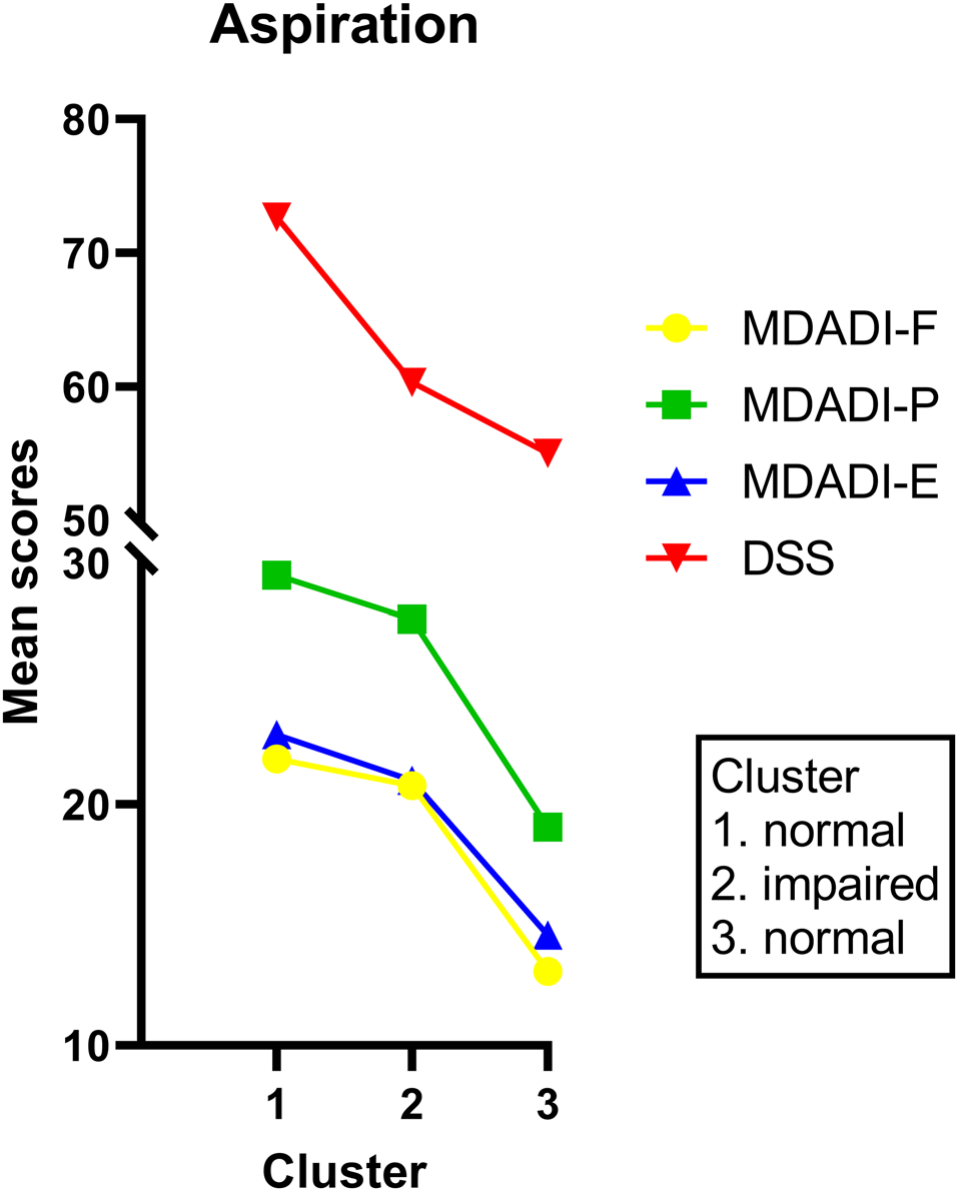
Mean MDADI subscale and DSS scores per patient cluster for the clinical patient label *aspiration* (presence of penetration or aspiration) [30]

Figure 2 shows the three clusters of patients for the clinical patient label *aspiration*. Cluster 1 (58%; *n* = 45/78) and cluster 3 (18%; *n* = 14/78) contain patients who did not present penetration and/or aspiration during FEES and/or VFS. Cluster 2 (24%; *n* = 19/78) consists of patients presenting penetration and/or aspiration. The mean MDADI subscale and DSS scores are listed in Table 3.

For the patients without signs of penetration and/or aspiration (clusters 1 and 3), the mean MDADI subscale (*p* < 0.001) and DSS (*p* = 0.041) scores were significantly different between cluster 1 and 3. Although these two patient clusters have similar scores on the IROMs, the mean scores of the PROMs of patients in cluster 1 were significantly higher (higher swallow-specific QoL) compared to patients in cluster 3 (Fig. 2).

No statistically significant differences in the mean MDADI subscale (*p* > 0.176) and DSS (*p* = 0.155) scores were found between patients with signs of penetration and/or aspiration (cluster 2) and patients from cluster 1 without these signs of OD. This means that although the scores on the IROMs were significantly different between cluster 1 and 2, the mean scores of the PROMs were similar.

For cluster 3 however, the mean MDADI subscale scores were significantly different compared to the scores of patients in cluster 2 (*p* < 0.001). The mean DSS scores did not significantly differ between patients in cluster 2 and cluster 3 (*p* = 1.000). So, although patients in cluster 3 did not present signs of penetration and/or aspiration, their mean MDADI subscale scores were significantly lower (lower swallow-specific QoL) compared to patients who did have signs of penetration and/or aspiration (cluster 2).

## Discussion

The objective of the present study was to determine the relationship between PROMs and IROMs on OD in PD. Only a relationship with a moderate agreement (AUC = 0.6–0.7) between the PROMs and IROMs on OD in PD was found. This suggests that there is some sort of inconsistency between the signs of OD identified by clinicians using FEES and/or VFS and patient self-report swallow-related QoL questionnaires.

This inconsistency between PROMs and IROMs is not new in the literature on neurogenic dysphagia. In a cohort of 119 PD patients, Nienstedt et al. found that only 50% of the patients with severe aspiration (Penetration Aspiration Scale score > 6 [30]) during FEES reported swallowing complaints in the relevant domains of the Unified Parkinson Disease Rating Scale (UPDRS) II and in the non-motor symptoms questionnaire (NMS). The majority of these patients described their difficulties as ‘slight restrictions in swallowing’ [32]. Pflug et al. used a single question to evaluate whether PD patients experienced swallowing impairment and compared this outcome to signs of OD using FEES [33] Only 6% (*n* = 5/119) of the PD patients showed a normal pharyngeal swallow during FEES. However, 73% (*n* = 87/119) denied any swallowing impairment. The majority of the PD patients without OD complaints showed pharyngeal pooling of dyed water (52%; *n* = 45/87), bread (93%; *n* = 81/87), and biscuit (86%; *n* = 75/87) and 16% (*n* = 14/87) showed aspiration [33]. Only 12–27% of the PD patients with signs of swallowing impairment during FEES reported swallowing complaints [32, 33]. The current study also showed a moderate agreement between PROMs and IROMS in dysphagic PD patients. However, it is important to emphasize that previous studies described PROMS using OD symptom and FHS questionnaires and did not report on swallow-related QoL questionnaires.

To further elaborate this moderate agreement between PROMs and IROMs in the present study, a two-step cluster analysis was performed. It was hypothesized that there are clusters of patients with similar outcomes on FEES or VFS resulting in similar clinical patient labels, but with different outcomes on MDADI or DSS scores. The cluster analysis could help to understand why some PD patients with similar signs of OD during FEES or VFS have swallowing complaints and others don’t. Using the two-step cluster analysis, patients of cluster 1 in the *glossopalatal* label (Fig. 1) showed signs of OD during FEES and/or VFS and at the same time the lowest mean MDADI subscale and DSS scores, representing a poor swallow-specific QoL. However, the clinical patient label *glossopalatal* also contained patients of cluster 2 who had the highest mean MDADI subscale and DSS scores (highest swallow-specific QoL), and signs of OD on the IROMs. In the attempt to identify confounders that could predict the differences in the level of swallow-specific QoL presented by patients in cluster 1 and 2, patient characteristics were added to the analysis. However, the variables age, gender, H&Y scale, and the score on the other clinical patient labels could not be identified as confounders. The exact reason for the significantly different mean scores on the PROMs in patients with similar IROMs was therefore not found in this study.

A similar result was seen for the clinical patient label *pooling*. Only two clusters were found: one with signs of postswallow vallecular and/or postswallow pyriform sinus pooling and the other without. Interestingly, the mean MDADI subscale and DSS scores did not significantly differ between both clusters. Apparently, the level of swallow-specific QoL did not seem to depend on the presence or absence of pharyngeal pooling.

There are numerous hypotheses regarding the pathophysiology of OD in PD. Different sites in the nervous system may be affected [34]. A possible explanation for the inconsistency between PROMs and IROMs on OD in PD may be that the different sites of pathology in the nervous system may affect the swallowing function and the subjective perception of this in a different way. So, the phenotype of OD of an individual PD patient seems to encompass more than just the biomechanical swallowing function measured by IROMs. The OD phenotype includes the dimension of ‘the subjective perception of the swallowing disorder by the patient’ as well. PROMs and IROMs really seem to represent different dimensions of OD that together determine an OD phenotype in an integrated manner. The most well-known hypothesis of the pathophysiology of OD in PD is the lack of dopamine in the basal ganglia [35]. Functional magnetic resonance imaging (fMRI) studies in healthy subjects showed increased activation in parts of the basal ganglia namely the globus pallidus and putamen during swallowing [36]. Restoring the dopamine levels in these areas using dopaminergic medication or deep brain stimulation seemed to significantly improve swallowing in some PD patients [37]. However, several studies showed no significant improvements or worsening of OD using dopaminergic medication or deep brain stimulation, suggesting that there are different pathophysiological mechanisms in developing OD [34, 37]. Another site of pathology in PD are the non-dopaminergic pathways which might be affected by the development of Lewy bodies. Lewy bodies are abnormal aggregations of mainly alpha-synuclein proteins and are related to neuronal cell loss [38]. These Lewy bodies appear in the brainstem and cortex as PD progresses and were found in important pathways related to swallowing such as the dorsal nuclei of the glossopharyngeal and vagal nerve [39]. Lewy bodies were not only found in the central nervous system, but also in the enteric nervous system, and in the sensory and motor nerves of the pharyngeal wall [40, 41]. A possible hypothesis is that these different sites of pathology relate to different phenotypes of OD in PD, and require different diagnostic and therapeutic approaches.

Besides the different sites of pathology which may relate to different phenotypes of OD in PD, the occurrence of compensatory mechanisms may be another attribute to the different phenotypes. Some PD patients develop compensatory mechanisms that prevent them from having swallowing complaints [42]. Using magneto-encephalography (MEG) a shift in cortical activation during swallowing was found from the affected supplementary motor area to the lateral motor, premotor, and inferolateral parietal cortices in PD patients without clinical signs of OD. PD patients with clinical signs of OD did not show this shift on MEG [42]. Next to this compensatory shift in cortical activation several other compensatory strategies such as bolus modification and volume adjustment by taking smaller sips or bites may spontaneously be developed by PD patients [43]. This may improve patient’s self-perception of swallowing, and also the safety and efficiency of swallowing, but does not necessarily improve the biomechanics of their actual swallowing disorder.

Multiple reasons may underlie this moderate agreement between PROMs and IROMs on OD in PD. The absence of a support network, the level of cognitive impairment, or the presence of neurobehavioral conditions such as mood disorders or optimism may affect a patient’s perception of swallowing [44]. This study highlights that there are PD patients with similar FEES and/or VFS findings that cannot be lumped together under the same pathophysiological umbrella due to their differences in PROMs. This research has an important clinical relevance since it can give rise to differentiations in OD management for PD in the future.

## Limitations of the Study

The present study has some limitations. Deep learning methods such as MLP analysis require a sufficient amount of input data in order to give reliable outcomes. Although several techniques were used to improve the statistical power, analyses with larger sample sizes may result in different outcomes. Moreover, specific confounders responsible for different clusters of patients within the same clinical patient label could not be identified. Maybe if other confounders were used in the statistical analysis, other clusters or OD phenotypes might have come forward. Data on possible confounders such as the precise duration of PD were certainly considered but often not clear. Patients came from all over the Netherlands and their medical history was obtained from the referring neurologist. The letters did not always provide clarity about the date of onset of PD.

## Conclusion

In conclusion, the present study confirms inconsistencies between the signs of OD found using FEES and/or VFS and the burden of OD a patient may experience. There are PD patients with similar IROMs based findings that cannot be lumped together under the same pathophysiological umbrella due to their differences in PROMs. Since the exact origin of these differences is not fully understood, it seems appropriate for the time being to take into account the different dimensions of OD during the swallowing assessment so that they can be included in the patient-tailored treatment plan.
